# Burnei’s disease: teratological spondylolysis

**Published:** 2015

**Authors:** ST Gavriliu, RA Ghiță, T El Nayef, A Burnei, CR Olaru-Barbilian

**Affiliations:** * ”M. S. Curie” Children’s Clinical Emergency Hospital, Bucharest, Romania; ** Baneasa Hospital – “Regina Maria” Private Health Care Network Bucharest, Romania; *** “Elias” University Emergency Hospital, Bucharest, Romania; **** ”Carol Davila” University of Medicine and Pharmacy, Bucharest, Romania; ***** Colentina Clinic Hospital

**Keywords:** spondylolysis, vertebral body-vertebral arch shifting, dorsal spondyloschisis, absence of myelomeningocele or meningocele

## Abstract

Teratological spondylolysis is a pathological entity noted for the first time in the specialty literature by Gh. Burnei in “The Spine Journal”, in September 25, 2014.

This disease was described in a short presentation of the first case treated by the author.

The aim of this paper was to expose in a didactic manner the main characteristic aspects of Burnei’s disease: embryological, clinical, imaging and treatment data and also to make known this pathological entity with all its pathognomonic diagnostic elements.

This paper was based on data obtained after analyzing 2 cases of teratological spondylolysis: a 18-year-old patient with triple L3-L5 teratological spondylolysis with Pang 1 spinal dysraphism and a 1-year-old child with teratological spondylolysis and retrospondylolisthesis.

## Introduction

Spondylolysis is a discontinuity between different segments of the vertebral arches. It can be pedicular, isthmic or lamellar. The factors that may cause it are **teratogenic** factors, **stress** factors, trauma followed by fractures, pathological bone **fractures **(osteogenesis imperfecta, osteopetrosis, von Recklinghausen disease, etc.) and **degenerative** factors [**1**]. 

Teratological spondylolysis (TS) appears either due to the fusion failure of the chondrification/ ossification centers or due to the **agenesis** of these centers. Both processes are accompanied by the resorption of the mesenchymal tissue interposed between these centers or the one that generates these centers. It can be localized **retrosomatic**, between the vertebral body and the pedicle, **isthmic** or **retroisthmic**, on the lamellar level. 

**Embryology**

The spine forms from the notochord and the sclerotomes that surround the notochord, in a metameric segmentation manner, followed by chondrification and ossification. 

**Fig. 1:**
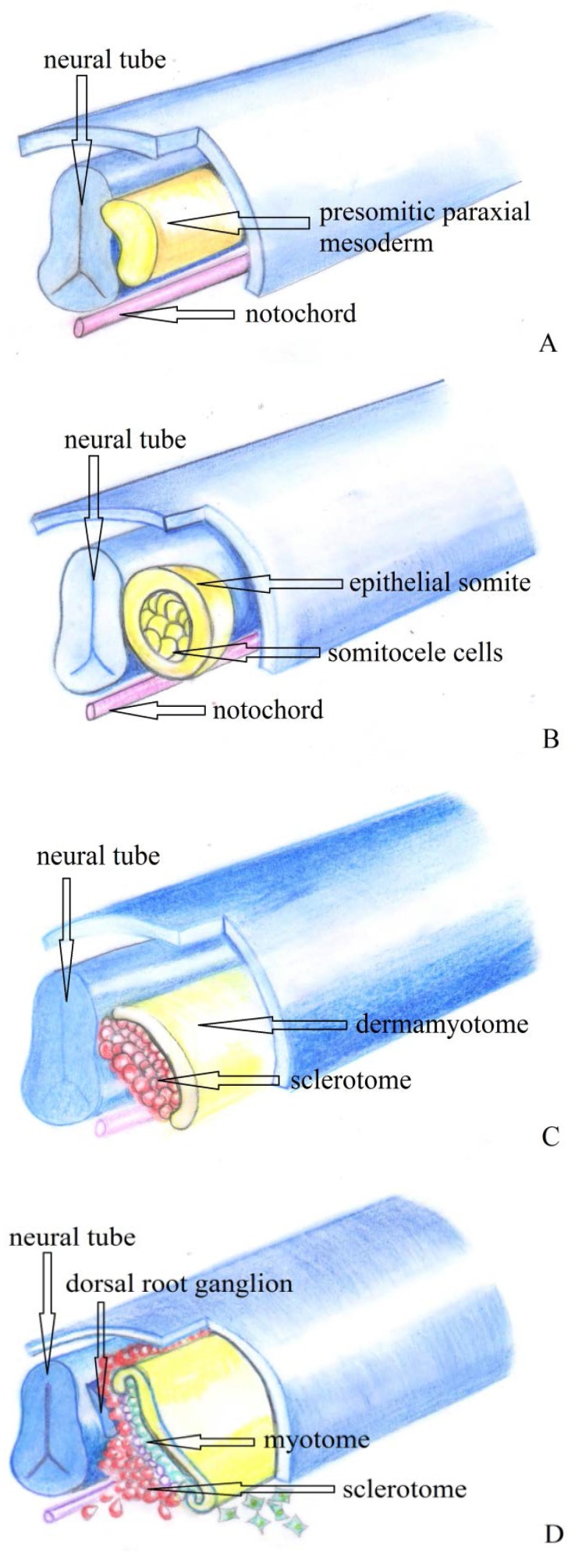
On each side of the neural tube the unsegmented paraxial mesoderm (presomitic) (A) forms segmented tissular blocks named somites (B) that lose their epithelial structure throw differentiation. Then, the somites differentiate into a ventro-medial portion (sclerotome) and a dorso-lateral portion (dermomyotome) (C). The sclerotome cells migrate around the neural tube and the notochord and from dermamyotome forms the myotome (D) (after Larsen’s Human Embryology)

The notochord appears out of the primitive streak in the 15th-16th day of gestation. It coordinates the normal segmentation of the blastomere. In the 4th week, it appears as a circular bar with giant cells (**Fig. 1A**). 

From the 5th week, the notochord degenerates and disappears leaving only the nucleus pulposus of the intervertebral disc. 

The sclerotomes are formed from the somitic paraxial mesoderm (**Fig. 1B**), like the dermomyotomes (dermatomes and myotomes) (**Fig. 1C**). The paraxial mesoderm disappears in the end of the 4th week; the cells of the sclerotome migrate towards and around the notochord (**Fig. 1D**); the closer ones form the perichondral sheath and the laterally placed ones form the origin of the vertebral bodies and intervertebral discs. 

**Fig. 2:**
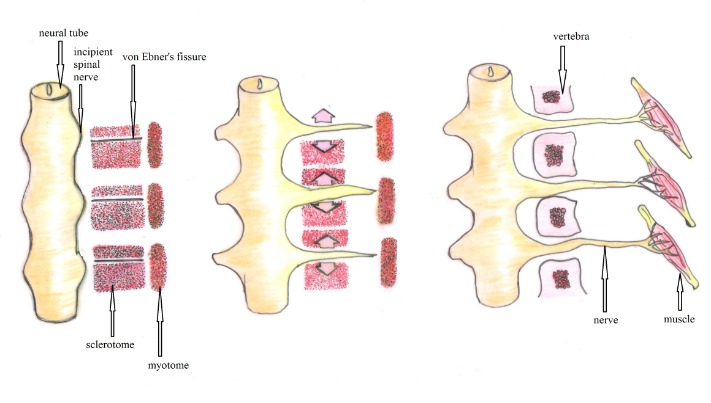
The sclerotome resegmentation throws out von Ebner’s fissure and the vertebral formation: the caudal half cells of a sclerotome proliferates and advances the throw of the intersegmental mesenchyme and interpolates with the cranial half cells of the bellow sclerotome; the myotomes join together the intervertebral discs and so we can move the vertebral spine (after Larsen’s Human Embryology)

Intersegmental arteries separate the sclerotomes, but a resegmentation of the sclerotomes appears after the formation of the Ebner fissure. Each vertebra forms by a merging of the cranial and caudal halves of 2 adjacent sclerotomes (**Fig. 2**). That is why the sclerotome and dermatome are not at the same level anymore.

The chondrification process starts in the 5th-6th week of gestation and ends in the 8th-9th week; the vertebral arches are open and get closed in the 17th week. The chondrification centers appear in the vertebral body and vertebral arches. The chondrification process can be altered by fusion failure of the chondrification centers or by the failure of formation of a bilateral center.

The ossification starts in the 12th week, when the embryo is 48 cm length. The ossification centers are separated by cartilaginous tissue and their fusion takes place around the age of 3-6 years.

**Fig. 3:**
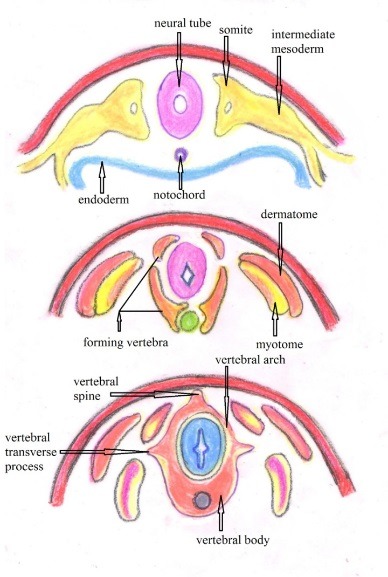
The sclerotome migration around the neural tube and the notochord forming the vertebral bodies, vertebral arches, vertebral transverse processes and vertebral spinous process (after Larsen’s Human Embryology)

From the 12th week until birth the cartilaginous tissue between the primary ossification center (of the vertebra) and the anterior-lateral primary center (of the pedicle) may undergo resorption determining the retrosomatic or somato-pedicular spondylolysis, the entire vertebral arch remaining free. The nonunion of the anterior primary center and the posterior one form the isthmic spondylolysis (**Fig. 3**); a part of the vertebral arch, the lamina, and the spinal process remaining free [**2**].

**Clinical data**

TS may be clinically silent. The “quiet” period is variable. It can stay asymptomatic throughout the entire life and therefore remain undiagnosed or diagnosed during necropsy if this is necessary for various reasons.

The symptoms appear in the first 20 years. The main **clinical signs** are a **radicular syndrome** or a **painful syndrome** established as spondylodiscitis [**3**].

The clinical signs are not typical. The paravertebral muscle contractures, limitation of flexion and lateral inclination of the trunk can appear [**4**]. Bony prominences are sometimes present, painful to local pressure or not. The bony prominences are given by the posterior vertebral arch or arches, which can be fixed by synchondrosis or synostosis to the cranial vertebral arches by the phenomenon of somato-sensitive shifting. The vertebral arches that remain in a floating status rarely form bony prominences. 

The diagnosis is exceptionally established at or immediately after birth. These patients may have other associated malformations (bladder exstrophy, lumbar agenesis, sacrococcygeal agenesis, agenesis of the femur, etc.) and their examination helps establish the diagnosis of TS.

**Imaging**

**Fig. 4:**
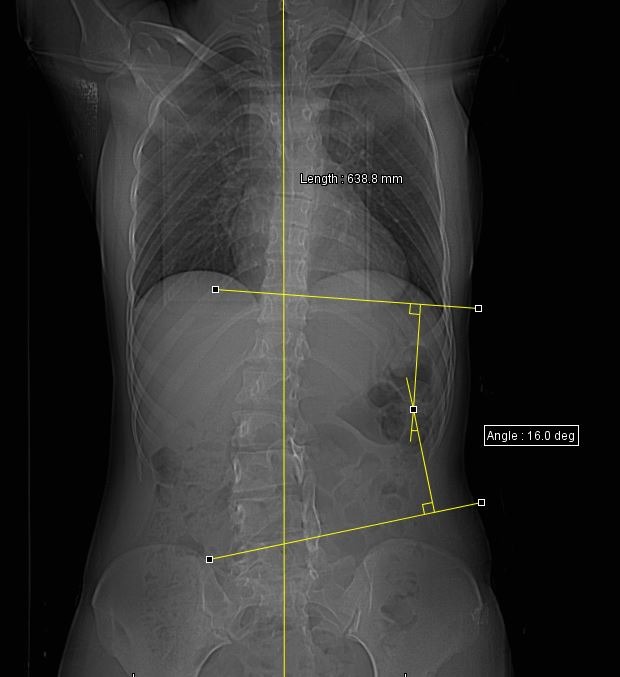
The posterior-anterior view shows a minimal scoliotic deviation of 16 degrees Cobb. On the left side, latero-somatic, a L3-L5 non-segmented bar is suggested

We were able to establish the right diagnosis only through imaging. On the lateral X-ray, the spondylolysis was seen as a discontinuity of the anterior margin of the vertebral bodies and the isthmic lysis. X-rays did not allow us establish a diagnosis of certainty. We were able to see a scoliotic curve and the presence of the bony masses, which might be the vertebral arches in the shifting position. These radiopaque images might erroneously suggest the presence of an unsegmented bony bar (**Fig. 4**).

**Fig. 5:**
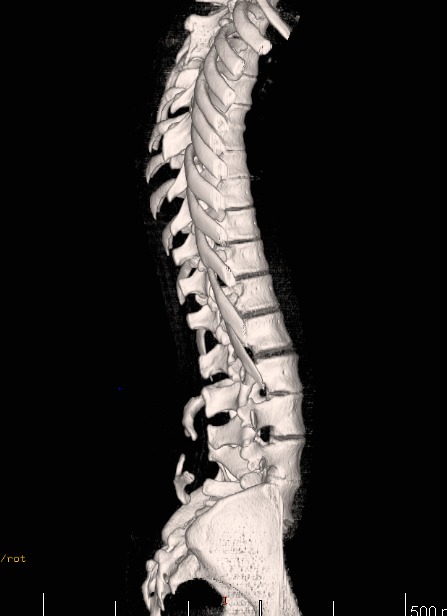
3D CT reconstruction in an 18-year-old patient: the lateral image reveals two vertebral arches arranged as crab forceps. L3 vertebral arch is positioned in a synchondrosis-shifting manner with the L2 spinous process. L4 vertebral arch is floating

The CT, 3D CT, and MRI are indispensable. The bony prominences are given by the posterior vertebral arch/ arches that are fixed by synchondrosis or synostosis to the cranial vertebral arches by the phenomenon of somato-sensitive shifting. The vertebral arches that remain in a floating status rarely form bony prominences (**Fig. 5**). 

**Fig. 6:**
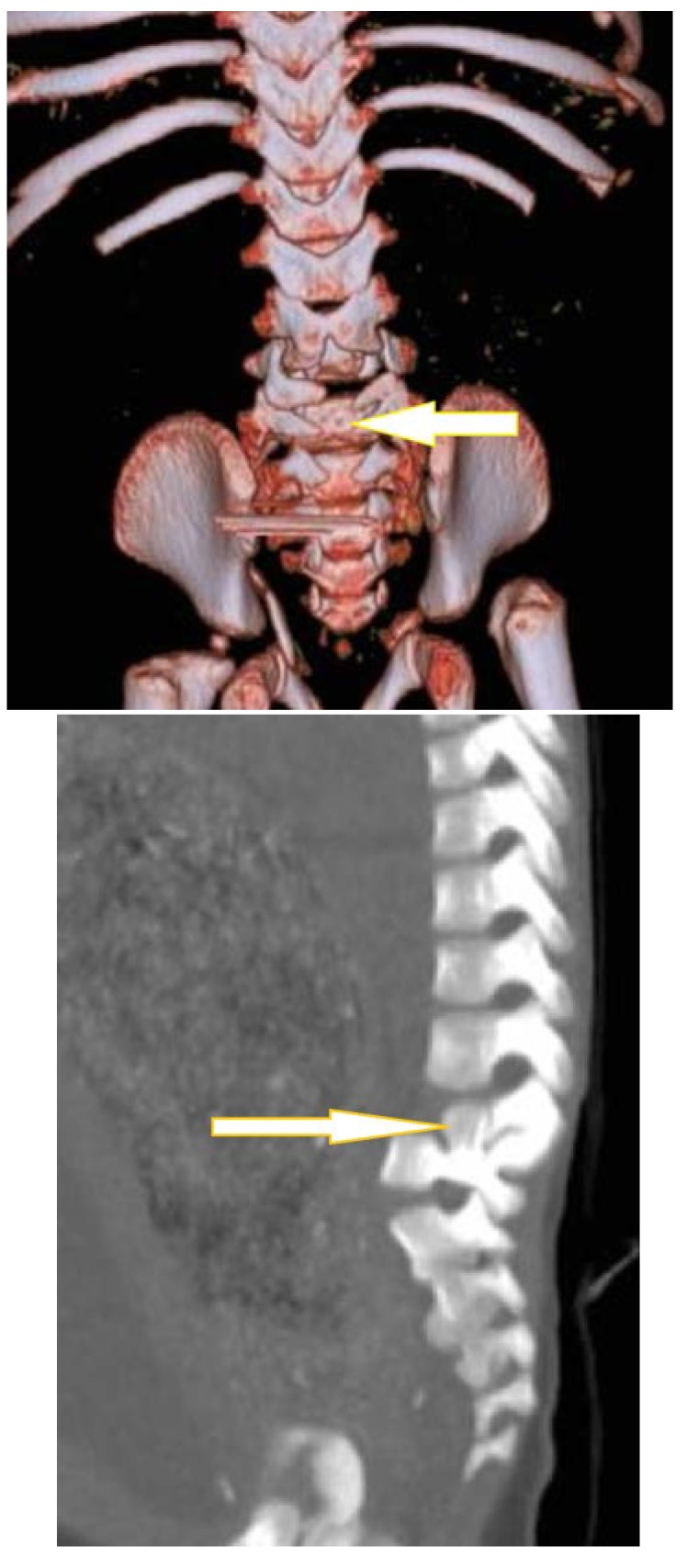
3D CT reconstruction in a 1-year-old patient with teratogenic spondylolysis: in coronal plane the image shows sacral spondyloschisis with left lamellar isthmic spondylolysis (left arrow) and the sagittal image highlights the posterior spondyloschisis with L3-L4 retrolisthesis (right arrow)

3D-CT, CT and MRI highlight the isthmic lysis dimensions, the hypoplastic grade of the vertebral arch, the body-vertebral arch discrepancy through the descensus body and ascensus arch process, as well as the associated malformations: diastematomyelia, segmentation or formation failure, spina bifida occulta, developmental hip dysplasia, etc. The dimensions of the spondyloschisis deformity are clearly reproduced by 3D-CT and CT (**Fig. 6**).

**Pathognomonic** for TS are the following: spondylolysis of one or more vertebral arches, vertebral arches shifting, dorsal spondyloschisis with the absence of myelomeningocele or meningocele.

An 18-year-old girl with triple L3-L5 teratological spondylolysis with Pang I spinal dysraphism and many other associated malformations had radicular syndrome and L4-S1 segmentation failure, which induced a 16-degree Cobb angle scoliosis [**5**]. 

**Treatment**

Surgery is not indicated if spondylolisthesis is not associated. The ablation of the arch is indicated if pain appears due to the synchondrosis or synostosis of the vertebral arch of the vertebra with spondylolysis and the above vertebral arch. Surgery is also indicated due to the esthetic point of view because the arch may form an undesirable prominence. If the floating arches are not painful, they should not be resected.

In evolution, in the first or second decade, a radicular syndrome or the hypoplasia of a limb may appear as a result of the associated malformations: congenital stenosis of the radicular foramen, diastematomyelia, etc. In these cases, surgery must treat the cause: foraminal ostium remodeling, diastematomyelia excision, etc.

If the 4D ultrasound establishes the intrauterine diagnosis, the surgery on the newborn may prevent the spondylolisthesis.

Surgical treatment in TS with spondyloschisis represents a prophylactic indication for the prevention of spondylolisthesis.

In older children or adolescents, the spondyloschisis after TS is treated by techniques currently used in the treatment of other forms of spondylolysis. 

**Acknowledgment**

GTS is part of POSDRU/159/1.5/S/137390.

## References

[1] Standaert CJ, Herring SA (2000). Spondylolysis: a critical review. Br J Sports Med.

[2] Schoenwolf GC, Bleyl SB Larsen’s Human Embryology.

[3] Shen M, Razi A, Lurie JD, Hanscom B, Weinstein J (2007). Retrolisthesis and lumbar disc herniation: a preoperative assessment of patient function. The Spine Journal.

[4] Syrmou E, Tsitsopoulos PP, Msrinopoulos D, Tsonidis C (2010). Spondylolysis: a review and reappraisal. Hippokratia.

[5] Burnei G, Gavriliu TS, Vlad C, Japie EM, Ghiță R (2015). L3–L5 teratological spondylolysis with diastematomyelia and L4 radicular syndrome followed by spondyloschisis without myelomeningocele due to somatoarcuate shifting. The Spine Journal.

